# Sensitive and Accurate Quantification of Enterovirus-D68 (EV-D68) Viral Loads Using Droplet Digital PCR (ddPCR)

**DOI:** 10.3390/microorganisms12081502

**Published:** 2024-07-23

**Authors:** Cassandra S. Grizer, Zhaozhang Li, Joseph J. Mattapallil

**Affiliations:** 1 The Henry M. Jackson Foundation for Military Medicine, Department of Microbiology & Immunology, Uniformed Services University, Bethesda, MD 20814, USA; 2 Biomedical Instrumentation Center, Uniformed Services University, Bethesda, MD 20814, USA; 3 Department of Microbiology and Immunology, Uniformed Services University, Bethesda, MD 20814, USA

**Keywords:** EV-D68, enterovirus, ddPCR, respiratory, viral loads, viremia

## Abstract

Enterovirus-D68 (EV-D68) is a reemerging virus that has been associated with numerous outbreaks in children in the past 10 years. Most assays examining viral infection kinetics have relied on the use of quantitative RT-PCR (qRT-PCR) assays as an assay of choice. Though valuable, there are inherent limitations that introduce variability, thereby reducing its value when comparing results across the field. Unlike the qRT-PCR assay that uses a standard curve to determine the copy number of viral RNA, the droplet digital PCR assay (ddPCR) directly quantifies the absolute number of copies within a given sample, which in turn makes the assay highly sensitive and accurate. Here, we have developed an EV-D68-specific ddPCR assay that effectively quantifies EV-D68 RNA copies in both cells and supernatants within a dynamic range of 6.7 × 10^−3^ copies/μL to 1.2 × 10^4^ copies/μL of the sample. The assay was highly specific for a broad range of EV-D68 isolates (Fermon, US/MO/14-18947, US/MO/14-18949, US/KY/14-18953, USA/2018-23088, USA/2020-23336 and EV-D68-infected human nasal turbinate samples from the 2022 outbreak) without cross-reactivity to other viruses such as Enterovirus-A71 (EV-A71), Human Parechovirus (HPeV)-1 and -2, Coxsackievirus (CV)-B1, Human Coronavirus (HCoV)-NL63, SARS-CoV-2, Influenza-A and B, Rhinovirus, and Respiratory Syncytial Virus (RSV)-A2, which are known to cause infection in children. The assay was able to readily quantify EV-D68 in infected cells and supernatants along with nasal turbinate samples collected from children during the 2022 outbreak. Our results suggest that the assay can be readily translated to accurately quantify viral loads in tissues and body fluids such as plasma and lung or nasal aspirates.

## 1. Introduction

The past decade has seen the re-emergence of enteroviruses such as Enterovirus-D68 (EV-D68) as a major public health concern [1]. Numerous outbreaks have been reported predominantly in children below the age of 16, starting with a major outbreak in the United States (US) and worldwide in 2014. Infections in children under the age of 5 are associated with severe respiratory disease, leading to an increase in pediatric hospitalizations due to hypoxia and wheezing [2]. In a subset of children, EV-D68 infection has been associated with polio-like paralytic symptoms characterized by acute flaccid myelitis (AFM) and lower motor neuron deficits in the spinal cord gray matter characterized by sudden onset muscle weakness, particularly in the limbs, with compromised reflexes and decreased muscle tone, and the paralysis of respiratory muscles in the most serious cases leading to respiratory failure [3].

Enterovirus-D68 is a non-enveloped, positive-sense, single-stranded RNA virus and a member of the *Picornaviridae* family. However, unlike other enteroviruses that infect via the fecal–oral route, EV-D68 is acid-labile, usually disrupted in the gastrointestinal tract, and primarily causes respiratory infections. EV-D68 was first isolated from children with pneumonia in 1962. Before 2005, only 26 cases were reported worldwide, and 699 cases were reported between 2005 and 2012. A large outbreak of EV-D68 infections was reported globally in 2014, with 1395 confirmed cases in the US from August 2014 to January 2015, constituting the first major EV-D68 outbreak [1]. The number of cases reported is likely a gross underestimate of the actual number of infections worldwide, likely due to the limited availability of clinical testing at the time [4]. EV-D68 predominantly circulates during the summer to fall season between August and October in temperate climates [5]. Unlike other viral infections, EV-D68 outbreaks tended to display a biennial pattern of prevalence, with a significant increase in the number of reported cases in 2014, 2016, and 2018. No major outbreak was reported in 2020, likely due to COVID-19-related masking, social distancing, and other containment measures. Since lifting these restrictions, EV-D68 infections have surged in Europe and the US, with a major outbreak reported in 2022 [6].

Quantitative RT-PCR (qRT-PCR) assays have been the method of choice to quantify EV-D68 RNA loads in clinical samples and have been used as a surrogate assay to determine the viral loads in body fluids and tissues [7,8,9,10,11,12,13]. Even though qRT-PCR is the most-used and best-known method for EV-D68 quantification and detection [14], there are a number of inherent limitations that contribute to the low sensitivity and precision quantification of viral RNA [15]. The qRT-PCR assay relies on the use of either RNA or plasmid standards to generate a standard curve that is then used to extrapolate the quantity of viral transcripts within a specific sample. How effective this extrapolation is dependent on a precise quantification of the concentration and serial dilution of each standard that introduces a significant potential for errors that will influence the calculation of the copy numbers. Furthermore, the copy numbers within each reaction can vary depending on where the threshold is drawn during analysis. Lastly, standard curves have to be generated for each plate, which can contribute to a high level of inter-assay variability and low sensitivity, preventing the accurate quantification of lower copy numbers of viral transcripts present in a sample [16].

The limitations of reproducibility and low sensitivity associated with current qRT-PCR assays have spurred the development of third-generation droplet digital PCR (ddPCR) assays that combine the random distribution of individual DNA targets in a single water–oil emulsion droplet followed by PCR amplification and Poisson statistics to accurately determine the number of target DNA copy numbers per microliter of the sample. Droplet digital PCR is highly sensitive, as the positive amplification of a single droplet is sufficient to accurately determine the concentration of the target DNA within the sample. Unlike qRT-PCR assays, ddPCR does not require standards for calculating copy numbers and counts the absolute number of DNA copies present in the reaction, thereby overcoming a major limitation associated with qRT-PCR assays. Additionally, ddPCR is an end-point PCR, meaning that low-performing primers or other PCR components do not affect the quantification of target DNA within a reaction. Lastly, further advancements allow for the development of ddPCR assays that can readily detect up to six different targets within a specific sample with low sample volumes [17]. Here, we sought to develop a ddPCR assay to accurately quantify EV-D68 viral RNA transcripts in supernatants and cells as an alternate approach to the qRT-PCR assay. Our results demonstrate that ddPCR can effectively quantify EV-D68 viral loads in a range of sample types.

## 2. Materials and Methods

### 2.1. Primers and Probes

Enterovirus-D68-specific primers and probes were designed using Primer3 software version 4.1.0 [18,19,20]. The probe was labeled with FAM at the 5′ end and BHQ1 quencher at the 3′ end (LGC Biosearch Technologies, Petaluma, CA, USA). The primer/probe mix (12.5 μM) was serially diluted starting at a 1:2 dilution to determine the concentration of primers/probe that yielded the number of target template copies/μL while still maintaining a significant difference in fluorescence amplitude between positive and negative droplets. Optimal concentrations of primers/probes were used for all downstream optimization and experiments using cells and supernatants.

### 2.2. RNA Isolation and cDNA Preparation

Rhabdomyosarcoma (RD) cells were infected with EV-D68 (US/MO/14-18947; BEI Resources, Manassas, VA, USA) at an MOI of 0.1. Cell pellets and supernatants were collected at 0, 24, 48, and 72 h post-infection (PI). Total RNA from cells was harvested by Trizol (Invitrogen, Waltham, MA, USA) extraction followed by RNA cleanup using the RNeasy kit (Qiagen, Hilden, Germany) per the manufacturer’s instructions. QIAamp virus MinElute Spin kit (Qiagen) was used to extract viral RNA from collected supernatants per the manufacturer’s instructions. RNA was quantified using NanoDrop (Thermofisher Scientific, Waltham, MA, USA).

Isolated RNA was reverse transcribed using the SuperScript III Reverse Transcriptase Kit (Thermo Fisher Scientific) described previously [21,22] and as per the manufacturer’s instructions. Briefly, RNA was denatured in the presence of random hexamers and dNTPs for 5 min at 65 °C in a thermocycler and transferred to ice for 5 min. After cooling, 2 μL of 10× RT-PCR buffer, 4 μL of 25 mM MgCl_2_, 2 μL of DTT, 0.5 μL of RNase/DNase free water, and 0.5 μL SuperScript III Reverse Transcriptase were added to the reaction mix and transferred to a thermocycler under the following conditions: 25 °C for 10 min, 50 °C for 50 min, and 5 min at 85 °C, before a final hold at 4 °C. If not used immediately, cDNA was stored at −20 °C.

### 2.3. Specificity of EV-D68 Primers

To establish the specificity of the EV-D68 VP1-specific primers/probe designed in the laboratory, viral RNA was isolated from Enterovirus-A71 (EV-A71), Human Parechovirus (HPeV)-1 and 2, Coxsackievirus (CV)-B1, Human Coronavirus (HCoV)-NL63, SARS-CoV-2, Influenza A and B, Rhinovirus, and Respiratory Syncytial Virus (RSV)-A2, and a broad range of EV-D68 isolates (Fermon, US/MO/14-18947, US/MO/14-18949, US/KY/14-18953, USA/2018-23088, USA/2020-23336 and EV-D68-infected human nasal turbinate samples from the 2022 outbreak) using the QIAamp virus MinElute Spin kit as described previously [23,24] and reverse transcribed to cDNA and amplified using the EV-D68-specific primers (forward 5′-CCAGATCGAGAGCATCATCA-3′, reverse 5′-AGGGACCACACCAAGTTCAG-3′) designed in the laboratory. Primers specific for EV-A71 and CV-B1 (forward 5′-TAACCCGTGTGTAGCTTGG-3′, reverse 5′-ATTAGCCGCATTCAGGGGC-3′) [25], HPeV-1 and 2 (forward 5′-CTGGGGCCAAAAGCCA-3′, reverse 5′-GGTACCTTCTGGGCATCCTTC-3′) [26], HCoV-NL63 (forward 5′-CTGTTACTTTGGCTTTAAAGAACTTAGG-3′, reverse 5′-CTCACTATCAAAGAATAACGCAGCCTG-3′) [27], SARS-CoV-2 (forward 5′-GACCCCAAAATCAGCGAAAT-3′, reverse 5′-TCTGGTTACTGCCAGTTGAATCTG-3′) [28], and RSV-A2 (forward 5′-GCTCTTAGCAAAGTCAAGTTGAATGA-3′, reverse 5′-TGCTCCGTTGGATGGTGTATT-3′) [29] were used as positive controls. A Taqman SYBR Green Relative PCR assay (Thermofisher) using a 7500 Real-Time qPCR system (Applied Biosystems, Waltham, MA, USA) was used with the following conditions: 95 °C for 15 min followed by 40 cycles of 94 °C for 15 s, 55 °C for 30 s, and 72 °C for 32 s.

### 2.4. Real-Time qPCR Assay

A quantitative PCR assay was developed using previously established protocols. Briefly, plasmid standards encoding the partial sequences of EV-D68 VP1 genes spanning across the regions detected by the EV-D68-specific primers/probe described above were generated synthetically and, after sequence verification, cloned into a pUC57-Amp plasmid encoding a BamHI cut site (Aldevron, Fargo, ND, USA) and linearized using BamHI. The linearized plasmid standard was serially diluted 10x to generate a standard curve. The qPCR assay was performed using a 7500 Real-Time qPCR system (Applied Biosystems). Samples were set up in a 96-well PCR amplification plate along with plasmid standards and no-template controls (NTC) using the following conditions: 94 °C for 5 min, 50 cycles at 94 °C for 15 s followed by 60 °C for 60 s. A standard curve was used to determine the copy number/reaction.

### 2.5. Droplet Digital PCR Assay

The ddPCR assay was performed using a QX200 droplet digital PCR System (BioRad Laboratories, Hercules, CA, USA) per the manufacturer’s protocol. Briefly, a reaction volume of 22 μL for each sample was mixed with the optimized concentrations of EV-D68-specific primers and probe along with 2× Supermix (Bio-Rad Laboratories) and RNase/DNase free water. Samples were emulsified using probe oil (Bio-Rad Laboratories) and droplets were generated using the QX200 Droplet Generator (Bio-Rad Laboratories). The emulsified reaction mix was transferred to a 96-well ddPCR plate, sealed with a plate sealer, and placed in a C1000 thermocycler (Bio-Rad Laboratories) using the following conditions: initial denaturation at 95 °C for 10 min, 40 cycles at 94 °C for 30 s, and 60 °C for 1 min, with a final deactivation of the enzyme at 98 °C for 10 min and ending at 12 °C, with a temperature variation rate of 2.5 °C/s. To determine the optimal annealing temperature for the EV-D68-specific primers and probe, a temperature gradient (58 °C to 61.1 °C) was tested using the C1000 Thermocycler (Bio-Rad). Samples were run in duplicate at each of the stated temperatures. The optimal annealing temperature was determined by the greatest difference in fluorescent amplitude between positive and negative droplets. After PCR amplification of the droplets, the samples were analyzed using the QX200 Droplet Reader (Bio-Rad Laboratories). Collected data were analyzed using the QuantaSoft software version 1.7 and QX Manager 2.1 (Bio-Rad Laboratories) to determine the number of copies/μL of the target nucleic acid per well. Thresholds were determined manually for each experiment based on the fluorescence intensity of the no-template control (NTC) and positive controls and fluorescent droplets above the threshold were considered positive. NTCs were included in each experiment, which resulted in zero positive droplets. Unless otherwise specified, all samples were run in triplicates, and the results were reported as resultant averages. EV-D68 plasmids with a concentration of 10,000 copies/mL or EV-D68 RNA (US/MO/14-18947, 746.1 ng/μL cDNA) were used as a positive control in each experiment. Samples with fewer than 10,000 total droplets were discarded, and the experiment was repeated. The EV-D68 copies/μL of cDNA was determined by dividing the total copies/μL of each reaction calculated by the droplet reader with the volume of cDNA used in that reaction.

The limit of detection (LOD) was determined using a serially diluted EV-D68 plasmid, whereas three NTCs were quantified to determine the limit of blank (LOB). The LOD was defined as the lowest number of copies that could be consistently detected in 1 μL of cDNA and the LOB was determined as the number of positive droplets detected in triplicate NTCs. To establish precision, triplicate experiments were set up with positive control EV-D68 plasmids as independent replicates and tested for significance using One-Way ANOVA. A *p* < 0.05 was considered significant. To determine inter-plate variability, the EV-D68 plasmid standard was serially diluted and quantified using both ddPCR and qRTPCR assays, and the copy numbers/μL of cDNA were compared between the two assays. Simple linear regression was performed to determine the line of fit, and Spearman’s correlation was used to determine the correlation coefficient; a *p* < 0.05 was considered significant.

## 3. Results

### 3.1. Optimization of EV-D68 Primer and Probe Concentration for ddPCR

**Figure 1:**
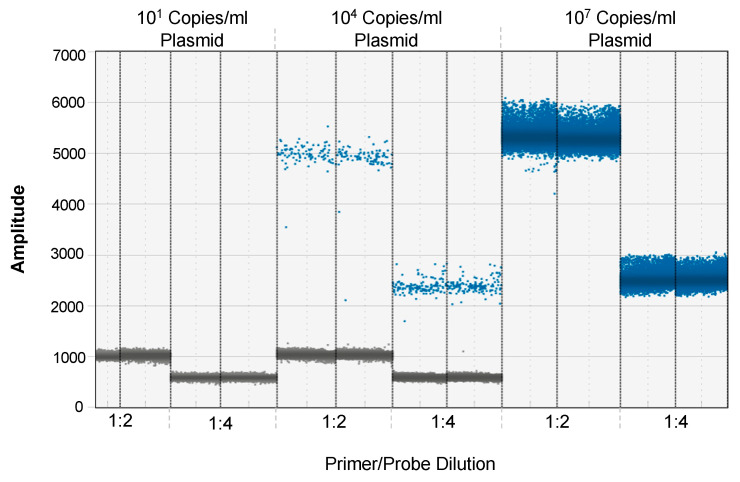
**Optimal concentration of EV-D68 primer/probe mix for ddPCR assay.** The primers/probe were diluted at 1:2 and 1:4. Representative dot plot showing the separation between the positive and negative droplets.

Excess primers and probes in a reaction mix have the potential to alter the signal-to-noise ratio. To overcome this concern, we first optimized the concentration of the EV-D68 VP-1-specific primers and probe for the ddPCR assay starting with the concentrations optimized for the qRT-PCR assay. Two dilutions of the primer/probe mix were tested using an equal amount of EV-D68 cDNA as the template in both dilutions. Though the separation between the positive and negative droplets at 1:2 dilution was higher than the 1:4 dilution, they were distinctly separated at the 1:4 primer/probe dilution to discriminate the positive droplets from the negative droplets (Figure 1). As the kinetics of viral loads decrease over time, the number of viral RNA copies declines after peak viremia. To detect these low copy numbers, a higher volume of the template needs to be included in the reaction mix. Using a 1:4 dilution of primer/probe mix compared to a 1:2 dilution permits the inclusion of additional templates without compromising the signal-to-noise ratio. Nonspecific amplification was not observed at either dilution, suggesting that a 1:4 dilution of primer/probe mix will optimally discriminate the positive from the negative droplets.

### 3.2. Optimal Annealing Temperature for ddPCR

**Figure 2:**
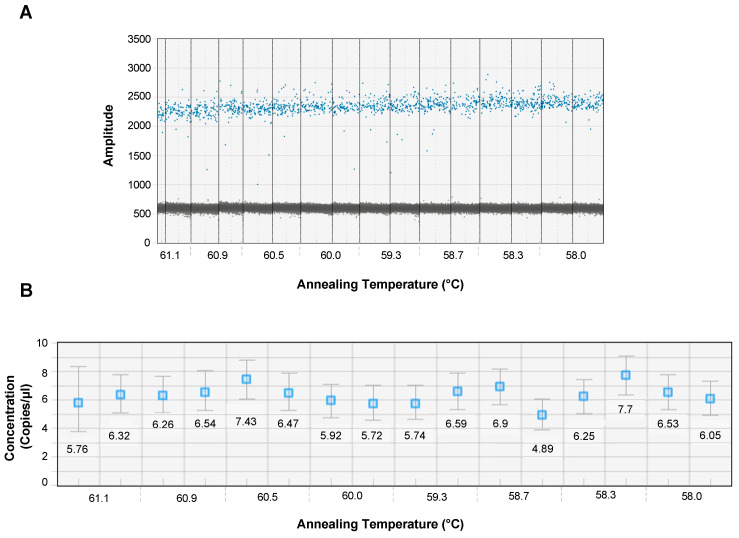
**Optimal annealing temperature for EV-D68 ddPCR.** The optimal annealing temperature for EV-D68 primers/probe was determined using a temperature gradient on a C1000 Touch Thermocycler. An EV-D68 plasmid standard with 10^4^ copies/mL was used as a template in duplicate at each temperature. (**A**) A dot plot showing the separation of positive and negative droplets by fluorescent amplitude at different annealing temperatures (58.0–61.1 °C) and (**B**) copies of EV-D68 per μL of the reaction mix at different annealing temperatures ranging from 58.0 to 61.1 °C.

An optimal annealing temperature is critical to clearly separate the negative from the positive droplets and avoid nonspecific annealing. To determine the optimal annealing temperature for EV-D68-specific primers/probe, we determined the optimal annealing temperature at a 1:4 dilution of primer/probe mix using an annealing temperature gradient at temperatures of 58.0, 58.3, 58.7, 59.3 60.0, 60.5, 60.9, and 61.1 °C using the C1000 thermal cycler. Our results showed that the fluorescent amplitude, droplet dispersion, and separation between positive and negative droplets (Figure 2A) and across different concentrations (Figure 2B) were readily detectable at an annealing temperature of 60 °C, which aligned with the annealing temperature optimized for the qRT-PCR assay.

### 3.3. Dynamic Range of the ddPCR Assay

**Figure 3:**
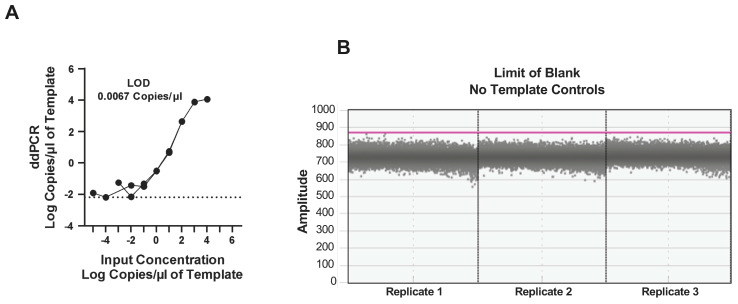
**Limit of Detection and Blank for ddPCR.** (**A**) Ten-fold dilutions of EV-D68 plasmid standard were analyzed to determine the upper and lower limits of detection (LOD). (**B**) Dot plot showing the separation of negative and positive droplets using no-template controls (NTC), in triplicate, to determine the limit of blank (LOB).

To assess the dynamic range, we determined the limit of detection (LOD) and blank (LOB) as these impact the sensitivity of the ddPCR assay. The LOD defines the lower and upper limits of the number of copies that can be detected in each reaction, whereas the LOB quantifies the number of false positives detected during the assay. The dynamic range for the assay was determined using an EV-D68 plasmid standard that was serially diluted to the lowest possible dilution to determine the maximum and minimum number of copies that could be detected using the ddPCR assay. Our results demonstrated that the highest number of copies that could be effectively detected without any loss of separation between the positive and negative droplets was 1.2 × 10^4^ copies/μL of cDNA. On the other hand, the lowest number of copies that could be detected was 6.7 × 10^−3^ copies/μL (Figure 3A). No false positive droplets were observed with triplicate NTCs, suggesting that the LOB was 0 copies/μL (Figure 3B). Taken together, our results indicate that the dynamic range for the assay is from 6.7 × 10^−3^ copies/μL to 1.2 × 10^4^ copies/μL of the sample.

### 3.4. Accuracy and Precision of the ddPCR Assay

**Figure 4:**
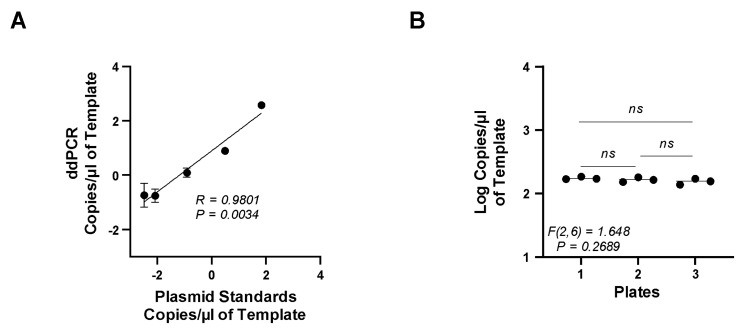
**Accuracy and Precision of ddPCR**. (**A**) EV-D68 plasmid standards were assayed with ddPCR. (**B**) Precision was determined using known plasmid standard in triplicate that was assayed on three separate plates. The copies/μL from each assay were compared with one-way ANOVA.

Accuracy assesses how close to the true copy number the assay can detect, while precision represents the repeatability of the assay. To determine the accuracy and precision of the EV-D68 ddPCR assay, we used the plasmid standards as unknown samples in the ddPCR assay and compared them to the known copies/μL of each standard used to derive the standard curve in the qRT-PCR assay using linear regression and Spearman’s correlation coefficient. Our results (Figure 4A) showed a significant positive correlation between the ddPCR assay and the known copy numbers (*R* = 0.9802, *p* = 0.0034).

To determine if the ddPCR assay results were precise and repeatable, multiple plates were tested with the exact same copies/μL of the positive control EV-D68 plasmid. These data were then compared across each plate to determine the degree of variability between the plates. Additionally, each positive control was also assayed in triplicate and compared using a one-way ANOVA. We found no significant difference in the number of copies/μL between each assay tested, suggesting excellent repeatability with little or no variability between the assays (Figure 4B).

### 3.5. Specificity of EV-D68 Primers

**Figure 5:**
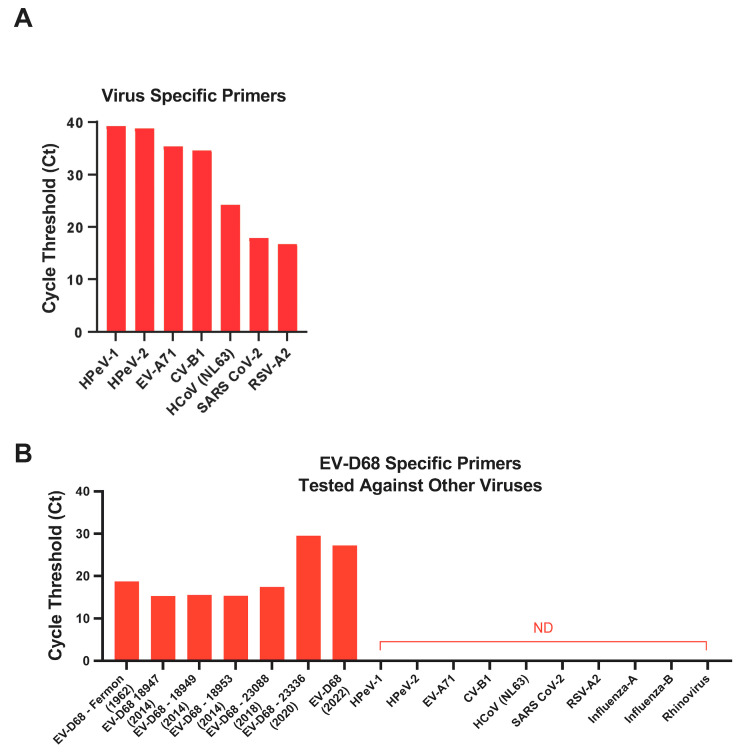
**Specificity of EV-D68 primers.** (**A**) The specificity of primers specific for HPeV-1 and -2, EV-A71, CV-B1, HCoV-NL63, SARS-CoV-2, and RSV-A2 were tested using RNA isolated from each virus in a SYBR Green relative RT-PCR assay. (**B**) The specificity of EV-D68 primers was tested against EV-D68 (Fermon, US/MO/14-18947, US/MO/14-18949, US/KY/14-18953, USA/2018-23088, USA/2020-23336 and an EV-D68-infected human nasal turbinate sample from the 2022 outbreak), HPeV-1 and -2, EV-A71, CV-B1, HCoV-NL63, SARS-CoV-2, RSV-A2, Influenza-A and -B, and Rhinovirus using EV-D68-specific primers in a SYBR Green relative RT-PCR assay.

To determine if the primers we designed in the laboratory were highly specific for EV-D68, we tested the specificity of the EV-D68 primers against a broad range of EV-D68 isolates (Fermon, US/MO/14-18947, US/MO/14-18949, US/KY/14-18953, USA/2018-23088, USA/2020-23336 and EV-D68-infected human nasal turbinate samples from the 2022 outbreak) and other human viruses, namely, EV-A71, HPeV-1 and -2, RSV-A2, CV-B1, HCoV-NL63, Influenza-A and B, Rhinovirus, and SARS-CoV-2, using relative SYBR green RT-PCR assay. HPeV-1 and -2, EV-A71, CV-B1, HCoV-NL63, SARS-CoV-2, and RSV-A2-specific primers were obtained from previously published studies and confirmed in a relative RT-PCR assay using RNA from each of these viruses (Figure 5A). Our results showed that EV-D68-specific primers only amplified the EV-D68 isolates but not the other viruses, demonstrating that the EV-D68 primers were highly specific and did not cross-react with the other viruses tested (Figure 5B).

### 3.6. Comparison of Real-Time qPCR and ddPCR Assays

**Figure 6:**
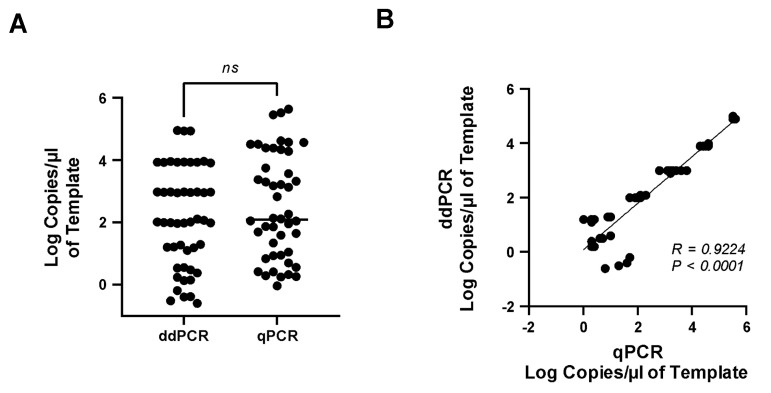
**Comparison and Correlation of qPCR and ddPCR.** Plasmid standard (10^7^ copies/mL) was serially diluted (ten-fold) and assayed using qPCR and ddPCR assays in parallel. (**A**) Number of EV-D68 copies/μL by ddPCR and qPCR assay. (**B**) Correlation of copy numbers derived from the ddPCR to the qPCR assay.

Real-time qRT-PCR assay is the most commonly used assay for detecting and identifying EV-D68 in clinical and research samples. To determine if copy numbers varied between the ddPCR and qPCR assays, the EV-D68 plasmid standard (10^7^ copies/mL) was serially diluted 10× and used as a template simultaneously in both assays. A paired *t*-test was used to determine if the number of copies significantly differed between the two assays. Our results showed that the number of EV-D68 copies detected by the ddPCR assay did not significantly differ from that of the qPCR assay (Figure 6A), with a significant positive correlation (*R* = 0.9224, *p* < 0.0001) between the two assays (Figure 6B). The ddPCR assay detected a significantly lower number of copies as compared to the qPCR assay (Figure 6A).

### 3.7. Quantification of EV-D68 Viral Loads in Cells, Supernatants, and Human Nasal Turbinates by ddPCR Assay

**Figure 7:**
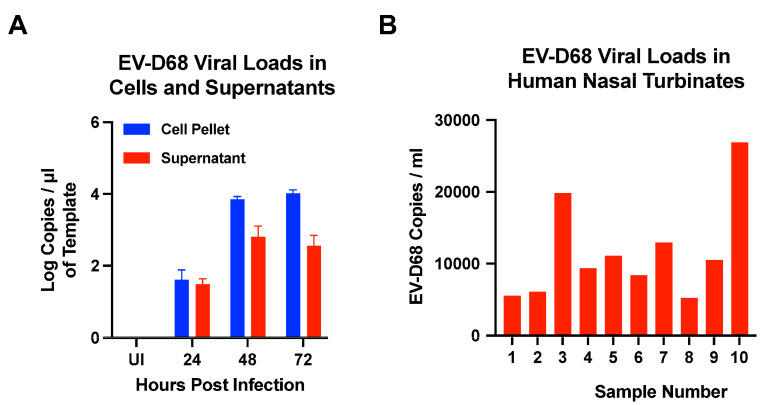
**Quantification of EV-D68 viral loads in cells, supernatants, and human nasal turbinate samples.** (**A**) Rhabdomyosarcoma (RD) cells were infected in triplicates with EV-D68 (US/MO/14-18947) at an MOI of 0.1. Cells and supernatants were collected at 24, 48, and 72 h post-infection. (**B**) EV-D68 viral loads in de-identified archival human nasal turbinate samples that were collected from children during the 2022 outbreak. EV-D68 viral loads were quantified using EV-D68-specific primers/probe by ddPCR.

To determine if the EV-D68-specific ddPCR assay we developed could effectively quantify both cell-associated and cell-free viruses, we infected RD cells with EV-D68 (US/MO/14-18947 isolate) at an MOI of 0.1 and harvested cells and supernatants collected at 0, 24, 48, and 72 h PI. Isolated RNA was reverse transcribed and used in the ddPCR assay for EV-D68. Our results demonstrated that both cell-associated and cell-free viruses could be readily detected at 24 h PI with viral loads peaking at 48 h PI (Figure 7). Interestingly, cell-associated viral loads were higher than cell-free viruses, suggesting that EV-D68 likely replicates to high levels in cellular compartments that remain sequestered inside the cells. Previous studies have reported that EV-D68 subverts autophagy to replicate leading to high levels of cell-associated viral loads as compared to the virus that is released into the supernatants [30]. Importantly, our results suggest that the ddPCR assay can be effectively used to quantify viral loads in both cells and supernatants.

We further validated the ddPCR assay by quantifying EV-D68 viral loads in de-identified archival human nasal turbinates that were collected from children during the 2022 outbreak. Our results showed that EV-D68 infection was readily detectable in the samples that were tested (Figure 7B), with viral loads ranging from 5248 copies/mL to 20,964 copies/mL of the sample demonstrating that the ddPCR assay could effectively quantify EV-D68 infection in human body fluids.

## 4. Discussion

The quantification of viral loads in experimental and clinical samples is a key endpoint in both in vitro and in vivo studies. Standard approaches rely on qRT-PCR assays to quantify viral RNA copies in various sample types, including both cell-associated and cell-free viruses [23,24,25,31,32]. Quantitative RT-PCR assays rely on the use of standards to generate a standard curve to extrapolate the total number of copies of viral RNA within a defined amount of sample that is a major source of inter- and intra-plate variability that impacts both the sensitivity and reliability of the assay, as has been reported in numerous studies [33,34,35]. The accuracy of the standard curve depends not only on the quantification of the exact copy number in each standard dilution but also on the technical skill of the end user in loading the standard dilutions into the PCR plate, both of which introduce significant levels of variability into the assay, which is compounded when processing a large number of sample sets which in turn compromise the reliability of the assay. Numerous other factors, such as the frequency of freeze–thaw cycles and mixing of the standards, etc., also influence the reliability and repeatability of the qRT-PCR assay [36]. The ddPCR assay, unlike the qRT-PCR assay, does not require the use of a standard curve and directly quantifies the absolute number of reverse transcribed RNA copies within a defined amount of sample. The total cDNA within each sample is emulsified in oil and separated into individual droplets, each of which carries 0–3 copies of the nucleic acid, which is then amplified in a thermocycler and quantified using a reader. Using this approach, we show that the EV-D68-specific ddPCR assay we developed could readily detect EV-D68 viral RNA as low as 6.7 × 10^−3^ copies/μL of the sample. The assay was highly specific from a broad range of EV-D68 isolates and samples without any apparent cross-reactivity with other viruses such as EV-A71, HPeV-1 and -2, RSV-A2, CV-B1, HCoV-NL63, SARS-CoV-2, Influenza-A and -B, and Rhinovirus, which are known to circulate and peak during the same season as EV-D68 [37,38,39,40,41,42], suggesting that the ddPCR assay we have developed can effectively discriminate EV-D68 infection from other seasonal viruses that are prevalent during the same time without false positive readout due to these viruses.

The quantification of viral loads is highly dependent on the number of viral RNA copies in the input template used in a ddPCR assay. As the kinetics of viral replication change over time, the levels of viral RNA significantly decline as compared to the peak of infection. In the case of positive-stranded RNA viruses, viral replication peaks between days 3–5 following infection, after which the viral loads decline to levels below detection by days 7–14 post-infection. As such, detecting viral RNA copies at low levels becomes challenging as the amount of input cDNA that can be added in a 22 ul ddPCR reaction mix is rather highly limited. We hypothesized that we could overcome this limitation by reducing the amount of primer/probe mix in the reaction. We tested this hypothesis by using the primer/probe mix at dilutions of 1:2 and 1:4. Our results demonstrated that even though the primer/probe mix at a 1:2 dilution gave a better separation between the negative and positive droplets, we could readily separate these droplets at a dilution of 1:4, thereby decreasing the total volume of the primer/probe mix we added to the sample and allowing that volume to be replaced with the template, which in turn increased the sensitivity of the assay to detect low copy numbers in samples.

The sensitivity of the ddPCR assay is significantly dependent on the specificity of the primer/probe binding accurately to the target template, which in turn depends on the annealing temperature. We initially optimized the primer/probe annealing temperature using the qRT-PCR assay and noted optimal annealing at 60 °C. To determine if the same annealing temperature would be optimal for the ddPCR assay, we set up a temperature gradient using annealing temperatures ranging between 58.0 and 61.1 °C on a C1000 Touch Thermocycler used for the ddPCR assay. Our results showed that an annealing temperature of 60 °C consistently amplified the target as compared to the other temperatures, suggesting that the same annealing temperature could be effectively used to directly compare the performance of both the qRT-PCR and ddPCR assays.

## 5. Conclusions

Taken together, our results show that the ddPCR assay is highly specific for EV-D68 without any cross-reactivity with the seasonal respiratory viruses that circulate among human populations at the same time. We were able to effectively quantify both cell-associated and cell-free EV-D68 RNA loads, suggesting that the assay could be readily translated to quantify infection in both tissues and body fluids such as plasma and lung lavages. The ddPCR assay overcomes a number of inherent limitations that are associated with the qRT-PCR assay and is able to effectively quantify EV-D68 RNA as low as 6.7 × 10^−3^ copies/μL of the sample, which would allow for more sensitive and precise quantification of EV-D68 infection in various sample types such as cells, supernatants and human body fluids like BAL, nasal washes and turbinates.

## Data Availability

The original contributions presented in the study are included in the article, further inquiries can be directed to the corresponding author.
